# Barriers and facilitators of health information seeking behavior among older adults: a mixed-methods systematic review using the COM-B model

**DOI:** 10.3389/fpubh.2026.1898275

**Published:** 2026-08-06

**Authors:** Endong Xie, Jianlin Ji, Liling Yang, Lili Chen, Wenxin Wu, Ouyao Chen, Chengyu Xing, Tanying Liao, Qunfeng Lu

**Affiliations:** 1 School of Nursing, Shanghai Jiao Tong University School of Medicine, Shanghai, China; 2 Department of Nursing, Shanghai Sixth People's Hospital Affiliated to Shanghai Jiao Tong University School of Medicine, Shanghai, China; 3 School of Nursing, Zunyi Medical University, Zunyi, China; 4 School of Nursing, Jiangxi University of Chinese Medicine, Nanchang, China

**Keywords:** barriers, facilitators, health information seeking behavior, older adults, systematic review

## Abstract

**Background:**

The intensification of global population aging has led to a growing demand for health information among older adults. Proactively seeking health information is crucial for enabling older adults to make informed health decisions. However, the factors influencing older adults' health information seeking behavior (HISB) have yet to be systematically summarized.

**Objectives:**

This study aims to identify the barriers and facilitators of HISB among older adults using the Capability, Opportunity, Motivation-Behavior (COM-B) model, and to provide a theoretical basis for developing intervention strategies.

**Methods:**

Following PRISMA guidelines, we conducted a search in seven databases, including PubMed, Embase, PsycINFO, CINAHL, Web of Science Core Collection, Scopus, and the Cochrane Library. The initial search period covered the period from the establishment of each database to 2 November 2025. The Mixed Methods Appraisal Tool was used to assess the methodological quality of the included studies. Researchers used convergent integration methods to collect, analyze, and integrate data. The COM-B model was applied to identify potential barriers and facilitators.

**Results:**

A total of 20 studies were included, comprising 16 quantitative descriptive studies, three qualitative studies, and one mixed-methods study. The findings highlight a complex and multifaceted range of factors influencing HISB, which were categorized into six domains: physical capability (skills to access information, age-related physiological decline and healthy lifestyle habits), psychological capability (limited ability to evaluate health information, health literacy and trust in the source of information), physical opportunity (credibility of accessible health information, access to information sources and financial difficulties), social opportunity (family support and social support), reflective motivation (self-efficacy, perceived usefulness of health information and intention to maintain health status), automatic motivation (personality trait).

**Conclusions:**

This review identified 15 factors influencing HISB among older adults and categorized them using the COM-B model. The analysis underscores the importance of addressing barriers and leveraging facilitators related to capability, opportunity, and motivation as a foundation for intervention design. Future research should further examine and validate these determinants and use them as a theoretical basis for developing targeted interventions to promote HISB among older adults.

**Systematic review registration:**

https://www.crd.york.ac.uk/PROSPERO/view/CRD42024600497, identifier: CRD42024600497.

## 1 Introduction

According to the latest report from the World Health Organization, the global population aged 60 years or older will increase from 1 billion to 1.4 billion between 2020 and 2030, reaching 2.1 billion by 2050 (1). This demographic shift has led to growing concerns about the health and wellbeing of older adults, who often face long-term health management challenges. As the prevalence of chronic conditions and multimorbidity increases with age, older adults are increasingly required to manage complex health information and make informed decisions regarding their care (2). Effective self-management therefore, relies on their ability to access, understand, and use relevant health information. Consequently, health information seeking behavior (HISB) plays a critical role in supporting informed decision-making and promoting better health outcomes among older adults (3).

Using grounded theory, Elizabeth et al. define HISB as a set of individual actions encompassing the seeking, acquiring, and verifying of health-related knowledge about specific health events or conditions (4). By enhancing health autonomy, HISB can improve health outcomes, boost patients' self-management capabilities, and thereby contribute to the achievement of healthy aging (5–7). Nevertheless, existing studies indicate limited engagement in HISB among older adults. Research by Cui et al. (8) and Hu et al. (9) demonstrates that older adults exhibited low levels of HISB. Furthermore, regarding the online dimension specifically, a multinational study involving 14,008 adults aged 60 years and older across 30 countries reported that only 49.22% had internet access and used it to seek health information, indicating limited engagement in health information seeking among this population (10). Addressing this challenge requires a comprehensive understanding of the factors that influence older adults' engagement in HISB, including barriers that hinder the initiation of information seeking behaviors and facilitators that support their continued involvement. Such an understanding is essential for developing targeted and effective behavioral interventions.

Existing research has identified a wide range of factors associated with HISB. For example, Mirzaei et al. (11) identified 67 categories of predictors, with age, education, gender, health status, and income emerging as key determinants. Other studies have explored factors influencing HISB in specific populations, such as cancer patients (12) and adolescents (13). While these studies provide valuable insights into the determinants of HISB, they do not comprehensively address the unique circumstances of older adults, whose health information seeking practices may be shaped by age-related changes, chronic disease burden, social support networks, and varying levels of access to information resources. Currently, only one review by Zhao et al. (14) has systematically examined factors influencing online health information seeking behavior among older adults, identifying personal, social, and information and communication technology-related barriers. Although this study provides important evidence regarding online information seeking, its exclusive focus on digital environments offers only a partial understanding of older adults' HISB. Older adults frequently obtain health information through multiple channels, including healthcare professionals, family members, friends, community networks, traditional media, and digital resources (15–18). Given the persistent digital divide experienced by many older adults, reliance on offline and interpersonal information sources remains common (19, 20). Therefore, restricting attention to online HISB may overlook important factors that influence HISB in later life. Moreover, existing studies have largely focused on listing potential influencing factors of HISB, while a comprehensive understanding of the barriers and facilitators that shape older adults' engagement in HISB remains limited. In addition, most previous studies lack the support of behavioral theoretical frameworks, which may hinder the translation of evidence into practical intervention strategies. Therefore, a theory-informed systematic synthesis is needed to comprehensively integrate barriers and facilitators influencing HISB across diverse information seeking contexts, thereby providing a deeper understanding of the complexity of HISB among older adults.

Numerous behavioral theories have been developed to explain behavior change; however, as highlighted by Michie and colleagues, selecting an appropriate theoretical framework from the extensive range of available theories can be challenging, with the risk of overlooking relevant theoretical perspectives and lacking a transparent rationale for theory selection (21). To address this challenge, Michie and colleagues developed the Capability, Opportunity, Motivation, and Behavior (COM-B) model by synthesizing constructs from 19 existing behavioral theories. The COM-B model incorporates both individual and contextual determinants of behavior and proposes that behavior results from the dynamic interactions among capability, opportunity, and motivation, thereby providing a comprehensive framework for categorizing behavioral determinants (22). Within this model, “Capability” encompasses the psychological and cognitive capacities for change; “Motivation” refers to the reflective and automatic mechanisms that energize and direct behavior; and “Opportunity” denotes the external factors that enable the behavior. Both capability and opportunity can directly influence behavior, and they can also indirectly affect it through motivation (23). The COM-B model has been widely applied to identify behavioral barriers and facilitators across diverse populations and behaviors, making it particularly suitable for understanding the multifaceted determinants underlying older adults' HISB (24, 25).

Therefore, guided by the COM-B model, this study aims to systematically synthesize the existing literature to identify and categorize the barriers and facilitators influencing HISB among older adults. As these determinants may be reported through both quantitative and qualitative evidence, we will conduct a mixed-methods systematic review to integrate findings from diverse study designs. This approach provides a rigorous methodological framework for developing a comprehensive understanding of the factors shaping older adults' HISB.

## 2 Methods

This review was reported following the PRISMA 2020 statement (26). The protocol was registered with PROSPERO in October 2024 (CRD42024600497). No amendments were made to the registered protocol.

### 2.1 Eligibility criteria

The inclusion criteria were as follows: (1) Type of study: qualitative, quantitative, or mixed methods research. Only published and peer-reviewed studies were included. Quantitative studies included factors influencing HISB in older adults; qualitative studies included the experiences or perspectives of older adults who engaged in HISB. (2) Type of participants: this review included adults aged 60 years or older, with no restriction on specific health conditions. (3) Type of outcomes: this review includes studies that have identified the barriers and facilitators of HISB among older adults using qualitative, quantitative, or mixed research methods. Barriers were defined as things that limit or prevent participation in HISB; facilitators were defined as things that make it easier to establish, reinforce, or sustain HISB (27). (4) Type of language: Only include research articles in Chinese or English.

### 2.2 Exclusion criteria

Exclusion criteria were: (1) factors influencing HISB were not explicitly reported in the study; (2) the publication was classified as gray literature, an editorial, letter, conference paper, case report, or abstract; and (3) the full text could not be obtained.

### 2.3 Information sources

We conducted a search in seven databases, including PubMed, Embase, PsycINFO, CINAHL, Web of Science Core Collection, Scopus, and the Cochrane Library. The search covered studies from database inception to 2 November 2025.

### 2.4 Search strategy

The search strategy includes subject and text terms for the following key concepts: aged, information seeking behavior. The search strategy was initially applied to PubMed and was subsequently modified based on the search requirements of other databases (Supplementary material 1).

### 2.5 Selection process

The search results were imported into the reference manager EndNote 20 to remove duplicates. Following this, two researchers (EX and JJ) each independently screened relevant articles based on title and abstract. The remaining literature from the screening was read in full text to finalize the literature for inclusion. If there was any disagreement during the screening of articles, it was first discussed and resolved by the two researchers, and if no agreement could be reached, a third researcher (LY) was added for counseling.

### 2.6 Data extraction

We strictly followed the developed inclusion and exclusion criteria, search strategy, and selection process. Two researchers (EX and JJ) independently extracted data using a standardized Excel spreadsheet and subsequently cross-checked the extracted information. The extracted data included the authors, year of publication, country, purpose of the study, study design, sample size, participant characteristics, and data collection. During the data extraction phase, barriers and facilitators, and mixed factors (which could not be distinguished as barriers or facilitators) were extracted from the final included literature.

### 2.7 Quality assessment

Two researchers (EX and JJ) independently used the Mixed Methods Appraisal Tool (MMAT) to assess the quality of the final included studies (28). The MMAT (Supplementary Material 8) can be used to assess the quality of five types of studies: qualitative, randomized controlled trials, non-randomized, quantitative descriptive, and mixed methods studies. In case of disagreement, the two individuals resolved the issue through discussion, and if disagreement remained, a third researcher (LY) was asked to participate in the consultation.

### 2.8 Synthesis methods

Convergent integrated approach was used to synthesize the data and integrate the findings according to the JBI methodology for Mixed Methods Systematic Review (29). Two researchers (EX and JJ) work independently on data integration. If they disagreed, the problem was resolved through discussion, and if disagreement remained, a third researcher (LY) was asked to participate in the consultation. The COM-B model was used to identify barriers and facilitators, and the following process was specified:

(1) Qualitative data analysis: two independent reviewers (EX and JJ) performed line-by-line coding of the qualitative findings using NVivo 11.0. Similar concepts were grouped together, and new codes were generated when necessary. The initial codes were then consolidated and refined to develop overarching themes (30). This process involved cross-checking to ensure the accurate capture and inclusion of relevant data within the themes.
(2) Quantitative data transformation: quantitative, non-textual data were transformed into qualitative data by two independent reviewers (EX and JJ) to facilitate integration with data extracted from qualitative studies and the qualitative components of mixed-methods studies. This process of “qualitizing” data involved converting numerical results into textual descriptions or narrative interpretations (29). The findings were then freely coded in NVivo 11.0 using an inductive approach, which were subsequently organized into themes. Cross-checking was also conducted in this phase.
(3) Data integration and theoretical mapping: the quantitative data were matched with the qualitative analysis results to further expand upon and deepen the understanding of the qualitative findings. Subsequently, the integrated results were mapped onto the COM-B model (22). The process of thematic mapping was a collaborative effort involving three researchers (EX, JJ, and LY). All identified themes were discussed and finalized by the entire research team. Any disagreements regarding the classification of themes within the COM-B domains were resolved through discussion.

## 3 Results

### 3.1 Study selection

**Figure 1:**
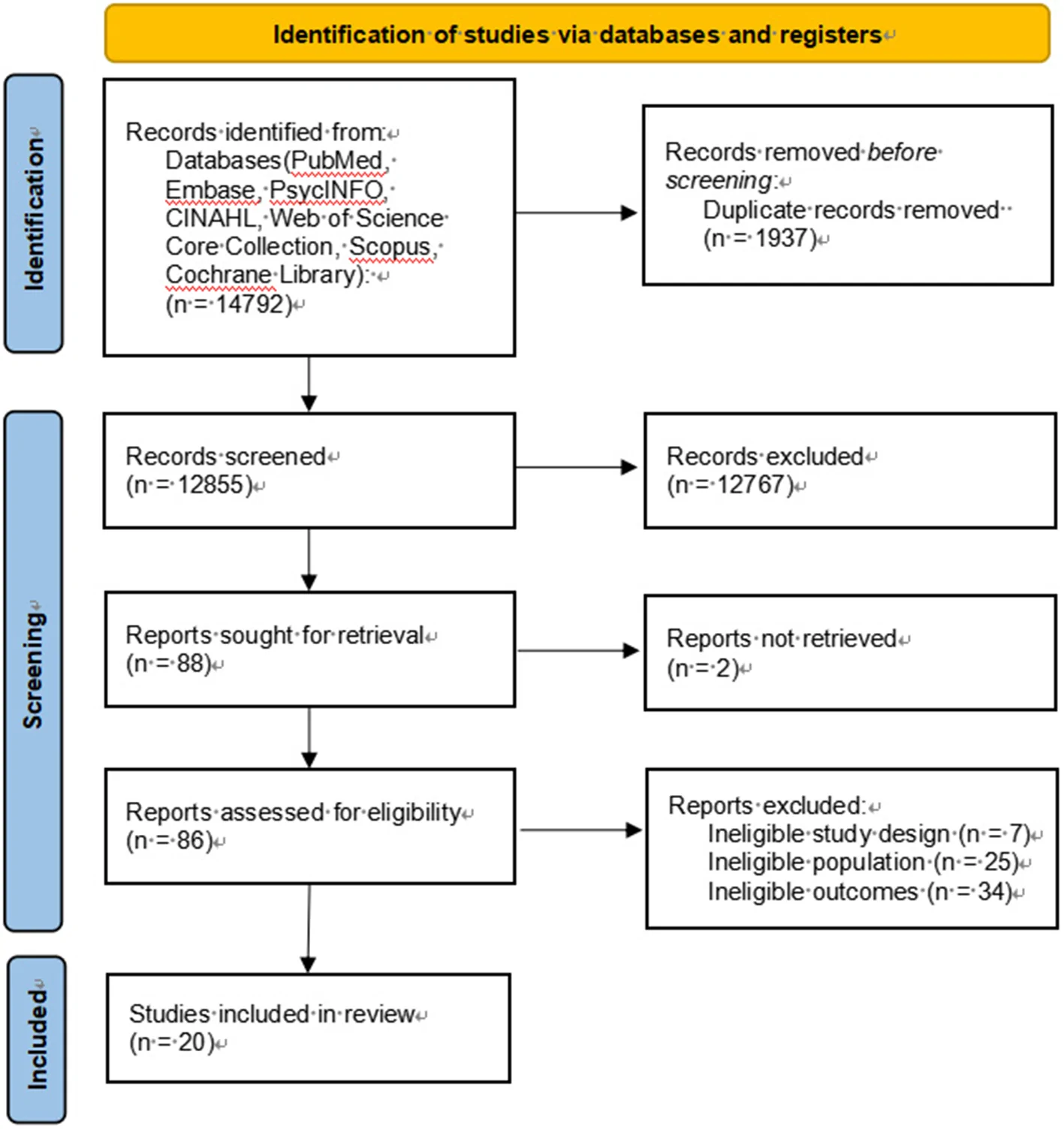
Study flow chart.

A search of the database yielded 14,792 records; removing duplicates yielded 12,855 records, and two researchers (EX and JJ) independently performed an initial screening of titles and abstracts, leaving 88 records that were considered potentially relevant. Finally, after full-text screening, 20 articles were included (Figure 1) for the extraction of barriers and facilitators.

### 3.2 Study characteristics

The catalog of all included studies is shown in Supplementary material 2, and the characteristics of the included studies are shown in Supplementary material 4. Of the 20 included studies, 16 were quantitative descriptive studies, three were qualitative studies, and one was a mixed methods study. The studies were conducted in eight countries, four of which were developed countries (*n* = 9) and four of which were developing countries (*n* = 11).

### 3.3 Risk of bias in studies

Most studies demonstrated robust methodology. Supplementary material 3 shows information on the quality assessment of the included studies. Eight studies met all five quality criteria of the MMAT, eleven 11 studies fulfilled four out of the five criteria, and only one study met three out of the five criteria. MMAT discouraged the exclusion of studies of lower methodological quality from its assessment and therefore included all of the studies for analysis.

### 3.4 Results of syntheses

Thematic analysis identified 15 themes for older adults and mapped them to six COM-B components (Table 1). Supplementary material 5 details the barriers and facilitators for each domain, along with the corresponding citations.

**Table 1:** Themes on facilitators, barriers, and mixed to HISB in older adults.

COM-B model component	Themes	Role	Study design
Physical capability	Skills to access information (9, 31, 32)	F	Quant/Qual
	Age-related physiological decline (8, 9)	B	Quant/Qual
	Healthy lifestyle habits (33, 34)	F	Quant
Psychological capability	Limited ability to evaluate health information (31)	B	Qual
	Health literacy (8, 34–37)	F	Quant
	Trust in the source of information (38–41)	F	Quant/Qual/Mixed
Physical opportunity	Credibility of accessible health information (8)	F	Quant
	Access to information sources (9, 36, 39, 42)	F	Quant/Qual/Mixed
	Financial difficulties (32, 36, 43)	B	Quant
Social opportunity	Family support (9, 31, 36)	F	Qual
	Social support (42, 44)	F	Quant
	Reflective motivation	Self-efficacy (8, 31, 42, 44, 45)	F	Quant/Qual
Perceived usefulness of health information (40, 41, 45)		F	Quant
Intention to maintain health status (31, 33, 35, 36, 38, 39, 41, 44, 46, 47)		F	Quant/Qual/Mixed
Automatic motivation	Personality trait (48)	M	Quant

F, facilitators; B, barriers; M, mixed factor; Quant, Quantitative; Qual, Qualitative; Mixed, Mixed methods.

### 3.5 Identified barriers and facilitators

The study identified 11 facilitators, three barriers, and one mixed factor, which highlights the complexity of HISB among older adults. These findings suggest that multiple influences should be considered when developing behavioral interventions.

#### 3.5.1 COM-B: capability (physical or psychological)

According to the COM-B model, capability is a prerequisite for behavior to occur. In this study, physical capability (skills to access information, age-related physiological decline, and healthy lifestyle habits) and psychological capability (health information discernment, health literacy, and trust in the source of information) were considered as key factors in this dimension.

Two facilitators and one barrier were identified in physical capability. Skills in accessing information (9, 31, 32) emerged as an important facilitator, as they enable older adults to locate and obtain relevant health information, thereby promoting engagement in HISB. In contrast, age-related physiological decline was identified as a major barrier (8, 9). For example, declines in memory, hearing, and cognitive functioning may impair older adults' ability to read, comprehend, and process health information, consequently hindering their HISB. Finally, healthy lifestyle habits (33, 34) were consistently associated with sustained engagement in HISB, indicating that health-conscious individuals are more likely to actively seek health information over time.

One barrier and two facilitators were identified in psychological capability. Older adults' limited ability to evaluate health information constrains their capacity to assess the quality and credibility of information, which in turn impedes their continued engagement in information seeking behaviors (31). Additionally, health literacy serves as a critical enabler of HISB, whereby individuals with higher literacy possess a greater capacity to source, comprehend, and utilize information when navigating intricate healthcare systems (8, 34–37). Finally, trust in the source of information emerged as an important psychological predictor of behavioral adoption (38–41), as older adults tended to place greater trust in health information delivered by healthcare professionals.

#### 3.5.2 COM-B: opportunity (physical or social)

In the COM-B model, opportunity implies external factors that promote behavior. This review identifies various factors in the opportunity dimension, including physical opportunity (credibility of accessible health information, access to information sources, and financial difficulties) and social opportunity (family support and social support).

The credibility of accessible health information (8) facilitated older adults' engagement in HISB by increasing their willingness to seek and use health information. Access to information sources (9, 36, 39, 42), including both online and offline channels, was also reported as a facilitator of HISB. In contrast, financial difficulties (32, 36, 43) acted as a barrier, limiting older adults' access to health information resources and reducing their engagement in HISB.

Two facilitators were identified in the social opportunity. Social support (42, 44) facilitated HISB by encouraging older adults to seek, discuss, and use health-related information. Family support (9, 31, 36) was also identified as a facilitator, with interactions and discussions with family members promoting older adults' attention to and engagement with health information.

#### 3.5.3 COM-B: motivation (re?ective or automatic)

The COM-B model emphasizes the importance of strong motivation in driving behavior. This study identified factors related to reflective motivation (self-efficacy, perceived usefulness of health information, and intention to maintain health status) and automatic motivation (personality trait).

Three facilitators were identified in reflective motivation. Self-efficacy (8, 31, 42, 44, 45) showed a positive correlation. Higher levels of self-efficacy are associated with greater self-awareness and more frequent engagement in seeking health information. Perceived usefulness of health information was a significant moderator of the Technology Acceptance Model, and research has found that it has a significant positive correlation with HISB (41, 45). Intention to maintain health status (31, 33, 35, 36, 38, 39, 41, 44, 46, 47) facilitated HISB, with health concerns and a desire to maintain or improve health motivating older adults to seek relevant health information. For example, individuals with a history of hyperglycemia were more likely to seek information related to diabetes prevention.

One mixed factor was identified in automatic motivation. Personality traits showed differential associations with HISB. Openness to experience was associated with greater engagement in health information seeking, whereas conscientiousness was associated with lower odds of seeking health information unrelated to physician visits. In addition, neuroticism was associated with increased information seeking before physician visits (48).

## 4 Discussion

### 4.1 Principal findings

To our knowledge, this is the first mixed-methods systematic review to comprehensively synthesize the barriers and facilitators of HISB among older adults. Consistent with previous studies, our findings identified a wide range of factors associated with HISB. However, unlike previous research that predominantly focused on digital-centric scenarios (14), the present study adopts a more inclusive perspective by examining HISB beyond independent online activities. By integrating both quantitative and qualitative evidence, this study successfully captures critical behavioral determinants that are uniquely salient to the aging population, such as trust in the source of information, ability to evaluate health information, and the supportive role of family and social networks. More importantly, the COM-B framework provided a theory-informed approach for synthesizing the evidence. By systematically mapping barriers and facilitators onto the domains of capability, opportunity, and motivation, this review contributes to a more structured understanding of how these factors shape older adults' HISB. Consequently, these synthesized insights not only clarify key behavioral entry points but also establish a robust theoretical blueprint for designing tailored interventions.

#### 4.1.1 COM-B: capability

In terms of physical ability, skills in accessing information were identified as an essential prerequisite for HISB. Older adults who lacked the skills to obtain health information, particularly through digital technologies, were less likely to engage in HISB, whereas those with stronger information-searching abilities reported greater confidence in meeting their information needs (9, 32). While the evidence synthesized in this review largely conceptualized such skills in terms of digital competencies, reflecting the increasing digitalization of health information environments, the growing adoption of smartphones among older adults does not automatically enable effective health information access; many remain confined to basic communication functions and require additional support to use these devices for health-related purposes (49, 50). Intervention studies confirmed that digital literacy training can improve both eHealth literacy and online HISB among older adults (51). Furthermore, the prominent role of family members and healthcare professionals identified in this review also indicates that older adults frequently rely on interpersonal channels for health information. Future research should therefore adopt a broader perspective on information-access skills and examine how older adults navigate and utilize multiple sources of health information across different contexts. Correspondingly, interventions may benefit from strengthening older adults' ability to access and use health information across both digital and interpersonal channels. Alongside these skill-based challenges, age-related declines in cognitive, sensory, and functional abilities may further impede HISB (8, 9), underscoring the need for age-friendly intervention design. Simplified interfaces, accessible educational materials, and emerging technologies such as voice-assisted systems may help reduce functional barriers and improve access to health information among older adults (52).

In terms of psychological ability, the ability to appraise health information, health literacy, and trust in information sources were identified as significant factors. Unlike younger populations, older adults often face challenges in evaluating the relevance, accuracy, and credibility of health information, particularly in increasingly digital information environments (53, 54). Limited ability to evaluate health information may make it difficult for older adults to distinguish reliable information from misinformation, thereby reducing their confidence and willingness to seek health information. This finding highlights that promoting HISB requires not only improving access to information but also strengthening older adults' capacity to critically evaluate the information they encounter. Regarding trust in information sources, four studies focused on older adults' trust in healthcare professionals (38–40), and only one study focused on older adults' trust in health information websites (41). Evidence suggested that trust may shape not only whether older adults seek health information, but also where they seek it. For example, older adults who reported positive communication with healthcare professionals were more likely to rely on these trusted sources for health information (38), whereas negative healthcare experiences could undermine trust and discourage information seeking (40). These findings underscore the importance of fostering trustworthy information environments and strengthening communication between older adults and healthcare providers to support informed HISB.

#### 4.1.2 COM-B: opportunity

In terms of physical opportunities, we finally summarized into three influencing factors, namely credibility of access to health information, access to information sources, and financial difficulties. Perceived credibility of health information appeared to facilitate HISB, as older adults were more willing to seek health information when they considered it trustworthy (8). However, the rapid expansion of digital health information has increased exposure to misinformation, making it challenging for older adults to identify reliable sources. Given the limited evidence on interventions aimed at improving information evaluation skills (55), future efforts should focus on enhancing the accessibility and trustworthiness of health information. Whereas access to information sources is a strong contributing factor, physicians are the most trusted source of information, but older adults perceive their lack of time as a barrier to meeting their information needs (39). Although the Internet may serve as an alternative source, usability barriers can reduce its effectiveness for older adults (9). Therefore, the management of information channels requires further strengthening. We can leverage offline social settings that are familiar to and relied upon by older adults as primary information gateways, thereby helping to mitigate the digital divide. Concurrently, providing simplified online tools as a supplementary measure can cater to the needs of diverse preference groups. Additionally, lower socioeconomic status may limit older adults' ability to access health information, potentially negatively impacting their health outcomes (32, 36). This is further evidence that digital health is not reaching all older people equally (43). Consequently, governments and healthcare institutions should assume a leading role in establishing a foundation for health equity through top-level design. This involves strengthening support for vulnerable older adults populations by, for example, mandating all public healthcare facilities to provide free and easy-to-read health brochures on common diseases and installing educational bulletin boards. Furthermore, family physicians should be encouraged to integrate health literacy promotion as a fundamental component of primary public health services.

In terms of social opportunities, we categorized them into two influencing factors: family support and social support. Previous studies have shown that older adults often discuss their health concerns with family members, receive care and assistance through family interactions, and obtain health-related information via these channels (31, 36). Such support may help older adults overcome barriers related to limited information-access skills by providing alternative pathways for obtaining health information, thereby facilitating engagement in HISB. Beyond the family context, broader social support networks may also promote health information seeking by encouraging older adults to engage with available information resources and participate more actively in health management (44). Research found that older adults who perceived themselves as health opinion leaders in social networks were more likely to pay attention to health information they encountered on WeChat and actively seek health information on the platform (42). These findings suggest that health information seeking among older adults is embedded within a broader social context rather than being solely driven by individual capabilities. Family members, peers, and community volunteers can serve as trusted intermediaries who facilitate information acquisition, encourage information seeking, and provide emotional support throughout the process, particularly for older adults who experience difficulties obtaining health information independently (56).

#### 4.1.3 COM-B: motivation

In terms of reflective motivation, we identified three influences, which are self-efficacy, perceived usefulness of health information, and willingness to maintain health status. Among these, self-efficacy consistently emerges as a foundational psychological resource that shapes older adults' engagement in health behaviors, including health information seeking (44). Evidence from multiple studies suggests that higher self-efficacy is associated with greater confidence in disease management and more proactive information acquisition, underscoring its role as a stable driver of sustained engagement (8, 31, 42, 44, 45). Perceived usefulness of health information further reflects a cognitive appraisal of the value of digital resources. Older adults perceive the Internet as a valuable tool for obtaining needed information, and studies have shown that the perceived usefulness of the Internet can positively influence older adults' willingness to contribute to HISB (41, 45). In addition, willingness to maintain health status represents a proactive, goal-oriented motivational orientation that internalizes health responsibility. This motivation tends to be strengthened under perceived health threats, such as existing illness, disease history, family history, or reduced quality of life (36, 38, 39, 46, 47), indicating that reflective motivation is not only cognitively driven but also closely tied to perceived vulnerability and health risk awareness. Taken together, these findings suggest that interventions aiming to enhance older adults' HISB should move beyond improving access alone and instead target psychological and cognitive drivers of motivation. Specifically, strengthening self-efficacy, enhancing perceived usefulness through user-centered digital health design, and reinforcing health responsibility awareness may be effective strategies to promote sustained engagement in health information seeking among older adults.

#### 4.1.4 Implications for practice

This systematic review provides important insights into the factors that influence HISB among older adults. The findings highlight the complexity of this process, which is characterized by a multitude of barriers and facilitators spanning all components of the COM-B model. Consequently, targeted interventions are necessary to address these factors and improve health information seeking. Our results further argue against a one-size-fits-all approach, suggesting instead that strategies should be tailored to the specific capabilities, opportunities, and motivations of the older adult population. Furthermore, future research could build on these findings by applying the Behavior Change Wheel to systematically link the identified COM-B domains to appropriate intervention functions and Behavior Change Techniques (BCTs), thereby facilitating the development and evaluation of theory-informed interventions to improve HISB among older adults.

#### 4.1.5 Advantages and limitations

This review has several strengths. A comprehensive search strategy was applied, together with a convergent integration approach, to systematically triangulate evidence. The use of an established framework, the COM-B model, provides a structured understanding of the facilitators and barriers to older adults' HISB, thereby informing the systematic development of targeted interventions. In addition, most included studies were of relatively good methodological quality, which strengthens the reliability of the findings.

We acknowledge the limitations of this review. Language bias may exist, as only English- and Chinese-language publications were included. Publication bias is also possible because the gray literature was not searched. Although a mixed-methods synthesis was conducted following established guidelines, the predominance of quantitative surveys, many of which were not validated and relied on closed-ended items, may have limited depth and introduced response bias. Finally, while this review aimed to capture a broad range of determinants of HISB among older adults, the evidence base largely focused on online contexts, which may limit the generalizability of findings. Future research should investigate HISB across diverse information seeking contexts, including both digital and non-digital environments, to determine whether barriers and facilitators vary across settings. Qualitative approaches may further enrich these findings by exploring older adults' experiences and contextual challenges in different health information seeking processes.

## 5 Conclusion

In conclusion, this review has identified 15 factors influencing HISB among older adults, categorized using the COM-B model. This analysis underscores the importance of addressing barriers and leveraging facilitators related to capability, opportunity, and motivation as a basis for intervention design. The multiplicity of these factors precludes a one-size-fits-all solution. Therefore, future efforts should be directed toward developing flexible, adaptable, and widely deliverable support strategies that can be tailored to meet the diverse needs of the aging population.

## Data Availability

The original contributions presented in the study are included in the article/Supplementary material, further inquiries can be directed to the corresponding author.
