# Supplementary material for: Barriers and facilitators of health information seeking behavior among older adults: a mixed-methods systematic review using the COM-B model
Source: Front Public Health. 2026 Aug 6;14:1898275. doi: 10.3389/fpubh.2026.1898275 (PMC13489831; doi:10.3389/fpubh.2026.1898275)
Supplement: Supplementary file 1 [file Table_1.docx]

***Supplementary Materials***

[Supplementary material 1 The search strategies for each database	2](#_Toc21389)

[Supplementary material 2 The list for the included studies	5](#_Toc20085)

[Supplementary material 3 Quality assessment of the included studies	7](#_Toc6374)

[Supplementary material 4 Study characteristics	10](#_Toc12294)

[Supplementary material 5 Barriers and facilitators derived from studies	22](#_Toc20346)

[Supplementary material 6 The Behaviour Change Wheel	54](#_Toc10771)

[Supplementary material 7 Definitions of COM-B model components and examples	55](#_Toc30997)

[Supplementary material 8 Mixed Methods Appraisal Tool (MMAT) 2018	56](#_Toc4765)

[Supplementary material 9 PRISMA 2020 Checklist	57](#_Toc2448)

Supplementary material 1 The search strategies for each database

1. **PubMed:**

| **Number** | **Search items** |
| --- | --- |
| #1 | "aged"[MeSH Terms] |
| #2 | "senior citizen"[Title/Abstract] OR "elder*"[Title/Abstract] OR "old"[Title/Abstract] |
| #3 | #1 OR #2 |
| #4 | "Information Seeking Behavior"[MeSH Terms] |
| #5 | Information Seek*[Title/Abstract] OR information-seeking behavio*[Title/Abstract] OR health information seeking behavio*[Title/Abstract] OR ISB[Title/Abstract] OR HISB[Title/Abstract] |
| #6 | #4 OR #5 |
| #7 | (view*[Title/Abstract] OR perspective* OR experience*[Title/Abstract] OR perception*[Title/Abstract] OR barrier*[Title/Abstract] OR challeng*[Title/Abstract] OR facilitator*[Title/Abstract] OR understand*[Title/Abstract] OR enabler* OR belief*[Title/Abstract] OR attitude*[Title/Abstract] OR behavior*[Title/Abstract]) |
| #8 | #3 AND #6 AND#7 |

2. **Embase**

| **Number** | **Search items** |
| --- | --- |
| #1 | ('aged'/exp OR aged OR senior) AND ('citizen'/exp OR citizen) OR elder OR old |
| #2 | (((information AND seeking AND behavior OR information) AND seek* OR 'information seeking') AND behavio* OR health) AND information AND seeking AND behavio* OR isb  OR hisb |
| #3 | view* OR perspective* OR experience* OR perception* OR barrier* OR challeng* OR facilitator* OR understand* OR enabler* OR belief* OR attitude* OR behavior |
| #4 | #1 AND #2 AND #3 |

3. **PsycINFO**

| **Number** | **Search items** |
| --- | --- |
| #1 | TI (aged OR senior citizen OR elder* OR old ) OR AB ( aged OR senior citizen  OR elder* OR old) |
| #2 | TI (Information Seeking Behavior OR Information Seek* OR information-  seeking behavio* OR health information seeking behavio* OR ISB OR HISB ) OR  AB (Information Seeking Behavior OR Information Seek* OR information-seeking  behavio* OR health information seeking behavio* OR ISB OR HISB) |
| #3 | TI (view* OR perspective* OR experience* OR perception* OR barrier* OR  challeng* OR facilitator* OR understand* OR enabler* OR belief* OR attitude* OR  behavior*) OR AB (view* OR perspective* OR experience* OR perception* OR  barrier* OR challeng* OR facilitator* OR understand* OR enabler* OR belief* OR  attitude* OR behavior*) |
| #4 | #1 AND #2 AND #3 |

4. **CINAHL**

| **Number** | **Search items** |
| --- | --- |
| #1 | TI (aged OR senior citizen OR elder* OR old ) OR AB ( aged OR senior citizen  OR elder* OR old) |
| #2 | TI (Information Seeking Behavior OR Information Seek* OR information-  seeking behavio* OR health information seeking behavio* OR ISB OR HISB ) OR  AB (Information Seeking Behavior OR Information Seek* OR information-seeking  behavio* OR health information seeking behavio* OR ISB OR HISB) |
| #3 | TI (view* OR perspective* OR experience* OR perception* OR barrier* OR  challeng* OR facilitator* OR understand* OR enabler* OR belief* OR attitude* OR  behavior*) OR AB (view* OR perspective* OR experience* OR perception* OR  barrier* OR challeng* OR facilitator* OR understand* OR enabler* OR belief* OR  attitude* OR behavior*) |
| #4 | #1 AND #2 AND #3 |

5. **Web of Science Core Collection**

| **Number** | **Search items** |
| --- | --- |
| #1 | (TI=(aged OR senior citizen OR elder* OR old)) OR AB=(aged OR senior citizen OR  elder* OR old) |
| #2 | (TI=(Information Seeking Behavior OR Information Seek* OR information-seeking  behavio* OR health information seeking behavio* OR ISB OR HISB)) OR AB=  (Information Seeking Behavior OR Information Seek* OR information-seeking  behavio*O health information seeking behavio*OR ISB O HISB) |
| #3 | (TI=(view* OR perspective* OR experience* OR perception* OR barrier* OR challeng  OR facilitator* OR understand* OR enabler* OR belief* OR attitude* OR behavior*))  OR AB=(view* OR perspective* OR experience* OR perception* OR barrier* OR  challeng* OR facilitator* OR understand* OR enabler* OR belief* OR attitude* OR  behavior*) |
| #4 | #1 AND #2 AND #3 |

6. **Scopus**

| **Number** | **Search items** |
| --- | --- |
| #1 | TITLE-ABS-KEY ( aged OR senior AND citizen OR elder* OR old ) |
| #2 | TITLE-ABS-KEY ( information AND seeking AND behavior OR information AND seek* OR information-seeking AND behavio* OR health AND information AND seeking AND behavio* OR isb OR hisb ) |
| #3 | TITLE-ABS-KEY ( view* OR perspective* OR experience* OR perception* OR barrier* OR challeng* OR facilitator* OR understand* OR enabler* OR belief* OR attitude* OR behavior* ) |
| #4 | #1 AND #2 AND #3 |

7. **The Cochrane Library**

| **Number** | **Search items** |
| --- | --- |
| #1 | aged OR senior citizen OR elder* OR old |
| #2 | Information Seeking Behavior OR Information Seek* OR information-seeking behavio* OR health information seeking behavio* OR ISB OR HISB |
| #3 | view* OR perspective* OR experience* OR perception* OR barrier* OR challeng* OR facilitator* OR understand* OR enabler* OR belief* OR attitude* OR behavior |
| #4 | #1 AND #2 AND #3 |

Supplementary material 2 The list for the included studies

1. Cui P, Ni X, Zong M, Xin X, Jiang Y, Li X. Status and path analysis of influencing factors of the health information seeking behavior in elderly patients with chronic diseases. J Shanghai Jiao Tong Univ Sci. (2022) 42:805-12. doi: doi.org/10.3969/j.issn.1674-8115.2022.06.016.
2. Xu WB, Lu ZZ, Mu L. Factors influencing proxy online health information seeking among the elderly: A study from the perspective of the elderly with chronic illnesses. Technol Health Care. (2024) 32:861-71. doi: 10.3233/thc-230270.
3. Siamian H, Shahrabi A, Balaghafari A. The Information Needs and Seeking Behavior of Elderly Patients in Educational and Therapeutic Hospitals: Unveiling Barriers to Information Accessibility. J Nurs Midwifery Sci. (2024) 11. doi: 10.5812/jnms-137493.
4. Hu Y, Qiu X, Ji C, Wang F, He M, He L, et al. Post-stroke experiences and health information needs among Chinese elderly ischemic stroke survivors in the internet environment: a qualitative study. Front Psychol. (2023) 14:1150369. doi: 10.3389/fpsyg.2023.1150369.
5. Weber W, Reinhardt A, Rossmann C. Lifestyle Segmentation to Explain the Online Health Information-Seeking Behavior of Older Adults: Representative Telephone Survey. J Med Internet Res. (2020) 22:e15099. doi: 10.2196/15099.
6. Oh YS, Choi EY, Kim YS. Predictors of Smartphone Uses for Health Information Seeking in the Korean Elderly. Soc Work Public Health. (2018) 33:43-54. doi: 10.1080/19371918.2017.1391150.
7. Binbai S, Chiangkhong A. A Causal Model of Factors Affecting Health Information-Seeking Behavior of Muslim Older Adults in Pattani Province, Thailand. J Behav Sci. (2025) 20:61-74. doi: 10.69523/tjbs.2025.286236.
8. Wang YP, Ouyang P, Zhao YH, Lu LM, Liu HY, Dai C, et al. Health Information-Seeking Behavior in Older Adults with Vision Impairment Among Different Stages of Eye Care: A Cross-Sectional Comparative Study. Geriatrics (Basel). (2025) 10. doi: 10.3390/geriatrics10040087.
9. Eriksson-Backa K, Enwald H, Hirvonen N, Huvila I. Health information seeking, beliefs about abilities, and health behaviour among Finnish seniors. J Libr Inf Sci. (2018) 50:284-95. doi: 10.1177/0961000618769971.
10. Mehawej J, Mishra A, Saczynski JS, Waring ME, Lessard D, Abu HO, et al. Online health information seeking, low atrial fibrillation-related quality of life, and high perceived efficacy in patient-physician interactions in older adults with atrial fibrillation. Cardiovasc Digit Health J. (2022) 3:118-25. doi: 10.1016/j.cvdhj.2022.03.001.
11. Theis S, Schäfer K, Schäfer D, Jochems N, Nitsch V, Mertens A. The Relationship Between Individual Coping and the Need to Have and Seek Health Information Among Older Adults: Exploratory Mixed Methods Study. JMIR Hum Factors. (2021) 8:e15858. doi: 10.2196/15858.
12. Agyemang-Duah W, Arthur-Holmes F, Peprah C, Adei D, Peprah P. Dynamics of health information-seeking behaviour among older adults with very low incomes in Ghana: a qualitative study. BMC Public Health. (2020) 20:928. doi: 10.1186/s12889-020-08982-1.
13. Sheng X, Simpson PM. Health care information seeking and seniors: determinants of Internet use. Health Mark Q. (2015) 32:96-112. doi: 10.1080/07359683.2015.1000758.
14. Li WS, Chang L. Aging in cyberspace: Exploring health information acquisition among older WeChat users. Glob Media China. (2024) 9:279-302. doi: 10.1177/20594364231206640.
15. Sedrak MS, Soto-Perez-De-Celis E, Nelson RA, Liu J, Waring ME, Lane DS, et al. Online Health Information-Seeking Among Older Women With Chronic Illness: Analysis of the Women's Health Initiative. J Med Internet Res. (2020) 22:e15906. doi: 10.2196/15906.
16. Zhu Y, Song Y, Wang Y, Ji H, Wang D, Cai S, et al. Relationships among patient activation, social support and online health information seeking of community-dwelling older adults living with coronary heart disease. J Adv Nurs. (2023) 79:161-9. doi: 10.1111/jan.15428.
17. Pourrazavi S, Kouzekanani K, Asghari Jafarabadi M, Bazargan-Hejazi S, Hashemiparast M, Allahverdipour H. Correlates of Older Adults' E-Health Information-Seeking Behaviors. Gerontology. (2022) 68:935-42. doi: 10.1159/000521251.
18. Zhu T, Xu H. Status and influencing factors of diabetes information acquisition among rural elderly with pre-diabetes in Yiyang City, China: a cross-sectional study. BMJ Open. (2019) 9:e029938. doi: 10.1136/bmjopen-2019-029938.
19. Meischke H, Eisenberg M, Rowe S, Cagle A. Do older adults use the Internet for information on heart attacks? Results from a survey of seniors in King County, Washington. Heart Lung. (2005) 34:3-12. doi: 10.1016/j.hrtlng.2004.06.006.
20. Flynn KE, Smith MA, Freese J. When do older adults turn to the internet for health information? Findings from the Wisconsin Longitudinal Study. J Gen Intern Med. (2006) 21:1295-301. doi: 10.1111/j.1525-1497.2006.00622.x.

Supplementary material 3 Quality assessment of the included studies

| Author and year | Screening | | Qualitative studies quality criteria | | | | | Randomized controlled trials quality criteria | | | | | Non-randomized studies quality criteria | | | | | Quantitative descriptive studies quality criteria | | | | | Mixed methods studies quality criteria | | | | | Score | Total (%) |
| --- | --- | --- | --- | --- | --- | --- | --- | --- | --- | --- | --- | --- | --- | --- | --- | --- | --- | --- | --- | --- | --- | --- | --- | --- | --- | --- | --- | --- | --- |
|  | S1 | S2 | 1.1 | 1.2 | 1.3 | 1.4 | 1.5 | 2.1 | 2.2 | 2.3 | 2.4 | 2.5 | 3.1 | 3.2 | 3.3 | 3.4 | 3.5 | 4.1 | 4.2 | 4.3 | 4.4 | 4.5 | 5.1 | 5.2 | 5.3 | 5.4 | 5.5 |  |  |
| Sumai Binbai 2025 | √ | √ |  |  |  |  |  |  |  |  |  |  |  |  |  |  |  | √ | √ | √ | √ | √ |  |  |  |  |  | 5 | 100 |
| Yaping Wang 2025 | √ | √ |  |  |  |  |  |  |  |  |  |  |  |  |  |  |  | √ | √ | √ | √ | √ |  |  |  |  |  | 5 | 100 |
| Wenbo Xu 2024 | √ | √ | √ | √ | √ | × | √ |  |  |  |  |  |  |  |  |  |  |  |  |  |  |  |  |  |  |  |  | 4 | 80 |
| Hasan Siamian 2024 | √ | √ |  |  |  |  |  |  |  |  |  |  |  |  |  |  |  | √ | × | √ | √ | √ |  |  |  |  |  | 4 | 80 |
| Wenshu Li 2024 | √ | √ |  |  |  |  |  |  |  |  |  |  |  |  |  |  |  | √ | √ | √ | √ | √ |  |  |  |  |  | 5 | 100 |
| Yaru Zhu 2023 | √ | √ |  |  |  |  |  |  |  |  |  |  |  |  |  |  |  | √ | √ | √ | √ | √ |  |  |  |  |  | 5 | 100 |
| Yufan Hu 2023 | √ | √ | √ | √ | √ | √ | √ |  |  |  |  |  |  |  |  |  |  |  |  |  |  |  |  |  |  |  |  | 5 | 100 |
| Sara Pourrazavi 2022 | √ | √ |  |  |  |  |  |  |  |  |  |  |  |  |  |  |  | √ | √ | × | √ | √ |  |  |  |  |  | 4 | 80 |
| Jordy Mehawej 2022 | √ | √ |  |  |  |  |  |  |  |  |  |  |  |  |  |  |  | √ | √ | × | √ | √ |  |  |  |  |  | 4 | 80 |
| Peirong Cui 2022 | √ | √ |  |  |  |  |  |  |  |  |  |  |  |  |  |  |  | √ | × | √ | √ | √ |  |  |  |  |  | 4 | 80 |
| Sabine Theis 2021 | √ | √ | √ | √ | √ | × | √ |  |  |  |  |  |  |  |  |  |  | √ | × | √ | × | √ | √ | √ | √ | × | × | 3 | 60 |
| Winja Weber 2020 | √ | √ |  |  |  |  |  |  |  |  |  |  |  |  |  |  |  | √ | × | √ | √ | √ |  |  |  |  |  | 4 | 80 |
| Mina S Sedrak 2020 | √ | √ |  |  |  |  |  |  |  |  |  |  |  |  |  |  |  | √ | √ | × | √ | √ |  |  |  |  |  | 4 | 80 |
| Williams Agyemang-Duah 2020 | √ | √ | √ | √ | √ | √ | √ |  |  |  |  |  |  |  |  |  |  |  |  |  |  |  |  |  |  |  |  | 5 | 100 |
| Tingting Zhu 2019 | √ | √ |  |  |  |  |  |  |  |  |  |  |  |  |  |  |  | √ | √ | √ | √ | √ |  |  |  |  |  | 5 | 100 |
| Young Sam Oh 2018 | √ | √ |  |  |  |  |  |  |  |  |  |  |  |  |  |  |  | √ | √ | √ | √ | √ |  |  |  |  |  | 5 | 100 |
| Kristina Eriksson-Backa 2018 | √ | √ |  |  |  |  |  |  |  |  |  |  |  |  |  |  |  | √ | √ | × | √ | √ |  |  |  |  |  | 4 | 80 |
| Xiaojing Sheng 2015 | √ | √ |  |  |  |  |  |  |  |  |  |  |  |  |  |  |  | √ | × | √ | √ | √ |  |  |  |  |  | 4 | 80 |
| Kathryn E. Flynn 2006 | √ | √ |  |  |  |  |  |  |  |  |  |  |  |  |  |  |  | √ | √ | × | √ | √ |  |  |  |  |  | 4 | 80 |
| Hendrika Meischke 2005 | √ | √ |  |  |  |  |  |  |  |  |  |  |  |  |  |  |  | √ | √ | × | √ | √ |  |  |  |  |  | 4 | 80 |

Supplementary material 4 Study characteristics

| Author and year | Study location | Study design/  Theoretical framework | Participants | Aims | Instrument used |
| --- | --- | --- | --- | --- | --- |
| Sumai Binbai 2025 | Thailand | Qualitative studies / protection motivation theory (PMT) | N = 400 (180 Male, 220 Female)  Age: ≥ 60 years (Most participants were aged 60-69 years.)  Health status: not target specific disease populations | To develop and validate a causal model of factors influencing health information-seeking behavior (HISB) among Muslim older adults in Pattani Province, Thailand. | Data were collected using structured questionnaires and interview forms.  Health status: use a checklist of chronic and serious diseases  Health information-seeking behavior: eight items on motivation, sources, evaluation, and barriers, rated on a 5-point Likert scale  Health literacy: adapted from Srisaeng and Deenamchued (2019) and based on Sørensen et al. (2012) (20 items in four components: access, comprehension, analysis, and application)  Health perception: 14 items addressing severity, risk, self-efficacy, and barriers, also rated on a 5-point Likert scale  Health responsibility: five items on proactive health behaviors |
| Yaping Wang 2025 | China | Qualitative studies / none | N = 248 (99 Male, 149 Female)  Age: ≥ 60 years  Health status: patients who suffered presenting visual acuity (measured with spectacles or contact lenses if available) of <0.5 (decimal notation) | To investigate and compare the health information-seeking behavior (HISB) of older adults with visual impairment across different stages of eye care. | Self-reported overall health and internet access were queried. Information about visual impairment regarding categories (unilateral or bilateral), duration, and severity levels in the better and the worse eyes were extracted from the medical records.  The HISB questionnaire was initially developed by Zamani et al. (2014). |
| Wenbo Xu 2024 | China | Qualitative studies / none | N = 23 (11 Male, 12 Female)  Age: ≥ 60 years (mean age [67.87 ± 4.60] years)  Health status: patients who suffered from one or more chronic illnesses (based on the International Classification of Diseases-10 [ICD-10]) | To explore the influencing factors and their relative significance in proxy online health information (OHI) seeking among the elderly with chronic illnesses. | 23 semi-structured in-depth interviews (mean duration 20 minutes) |
| Hasan Siamian 2024 | Iran | Quantitative descriptive studies / none | N = 200 (97 Male, 103 Female)  Age: ≥ 65 years  Health status: patients hospitalised for more than three days | To investigate the information needs and seeking behavior of elderly patients in educational and therapeutic hospitals while also identifying the barriers that hinder these patients’ access to relevant information sources. | The questionnaire was designed by the researchers based on various Persian and English sources. |
| Wenshu Li 2024 | China | Quantitative descriptive studies / the theory of  motivated information management (TMIM) | N = 407 (96 Male, 305 Female, 6 Non-response)  Age: ≥ 60 years (mean age [68.54 ± 6.21] years)  Health status: not target specific disease populations | To explore the association between two cognitive factors (outcome ex-  pectancy and efﬁcacy) and two relational factors (social support and health opinion leadership) with  older WeChat users’ health information seeking and scanning behaviors. | The questionnaire was reformulated by the researcher based on the relevant entries.  Outcome expectancy :Three items adapted from Aﬁﬁ and Weiner (2004) and Chang (2014).  Efﬁcacy: Measures of efﬁcacy were adapted from Aﬁﬁ et al. (2006) and Li et al. (2018).  Social support: The questions were modiﬁed from Hajli (2014).  Health opinion leadership: Boster et al.’s (2011) scale  Health information seeking: Three items adapted from Ahadzadeh et al. (2018) were used to measure  participants’ frequency of seeking health information.  Health information scanning: Three items adapted from Ahadzadeh et al. (2018) and Waters et al. (2016) were used to test participants’ frequency of attending to health information. |
| Yaru Zhu 2023 | China | Quantitative descriptive studies / information-motivation-behavioural skills Model (IMB) | N = 451 (187 Male, 264 Female)  Age: ≥ 60 years (mean age [69.88 ± 6.43] years)  Health status: self-reported diagnosis of coronary heart disease | To explore the relationship between patient activation (subjective initiative of patients to participate in disease management in self-health care), social support, self-efficacy and online health information seeking among older patients with coronary heart disease. | Online health information seeking scale: the online health information seeking Scale (Wang 2019)  Patient activation: patient activation measure (Hibbard 2004)  Social support: the social support rating scale (Xiao 1994)  Self-efficacy: Chinese version of the general self-efficacy scale (Wang 2001) |
| Yufan Hu 2023 | China | Qualitative studies / none | N = 15 (8 Male, 7 Female)  Age: ≥ 60 years (mean age [64.67  ± 4.32] years)  Health status: have been diagnosed with an ischemic stroke for at least 1 month | To explore the experiences of older stroke patients when searching for health information, focusing on search methods, identification of health information, and difficulties faced during the search process. | 15 semi-structured interviews |
| Sara Pourrazavi 2022 | Iran | Quantitative descriptive studies / health belief model (HBM), technology acceptance model (TAM) | N = 320 (162 Male, 158 Female)  Age: ≥ 60 years (mean age [64.36  ± 4.41] years)  Health status: not target specific disease populations | To identify the potential motivators and probable barriers of e-health information-seeking behaviors (e-HISB) among older Iranian adults. | Most of the entries in the questionnaire were developed by the research team itself.  Self-Efficacy: a modified version of the information seeking self-efficacy scale (Bronstein J 2014)  Barriers to e-HISB: barriers to the e-health information seeking psychological scale (Kartiwi M 2019), barriers to the internet use scale (Chang J 2015) |
| Jordy Mehawej 2022 | USA | Quantitative descriptive studies / none | N = 874 (459 Male, 415 Female)  Age: ≥ 65 years (mean age 74.5 years)  Health status: patients with atrial fibrillations (AF) | To examine patient characteristics associated with online health information seeking and the association between information seeking and low AF-related quality of life and high perceived efficacy in patient-physician interaction. | Participants’ AF-related QOL: atrial fibrillation effect on quality of life questionnaire (Spertus J 2011)  Self-efficacy in patient-physician interactions: perceived efficacy in patient-physician interactions (Maly R.C 1998) |
| Peirong Cui 2022 | China | Quantitative descriptive studies / comprehensive model of information  seeking (CMIS) | N = 502 (223 Male, 279 Female)  Age: ≥ 60 years (mean age [68.98±5.59] years)  Health status: patients who suffered from one or more chronic illnesses (based on the International Classification of Diseases-10 [ICD-10]) | To understand the status quo, influencing factors and action paths of health information seeking behavior in elderly patients with chronic diseases. | Health literacy : health literacy management scale (Jordan J.E 2013)  Self-efficacy: chronic disease self-efficacy scale (Lorig K.R 2001)  Health information seeking behavior: Health information seeking behavior scale (Zamani M 2014) |
| Sabine Theis 2021 | Germany | Mixed methods studies / none | N = 26 (8 Male, 18 Female)  Age: ≥ 60 years (mean age [71  ± 7] years)  Health status: not target specific disease populations | To examine the relationship between individual coping and the need to have and seek health information among older adults. | 18 semi-structured interviews and answered the questionnaires  8 only answered the questionnaires  Individual coping: coping inventory for stressful situations (Endler N.S 1990)  Health information need / health information-seeking behavior: information need questionnaire (a specially created, theory-based questionnaire) |
| Winja Weber 2020 | Germany | Quantitative descriptive studies / none | N = 701 (313 Male, 388 Female)  Age: ≥ 60 years (mean age [71 .6 ± 7.3] years)  Health status: not target specific disease populations | To examine older people’s health-related internet use. | The questionnaire was developed by the researcher himself |
| Mina S Sedrak 2020 | USA | Quantitative descriptive studies / none | N = 72806 (only Female)  Age: ≥ 65 years  Health status: patients who suffered from one or more chronic illnesses | To describe online health information–seeking behavior among older patients with chronic illnesses and to compare the characteristics of patients who report using the internet to obtain health information with those who do not. | WHI Extension Study Supplemental Questionnaire (Form 156) |
| Williams Agyemang-Duah 2020 | Ghana | Qualitative studies / none | N = 60 (13 Male, 47 Female)  Age: ≥ 65 years  Health status: not target specific disease populations | To explores a disadvantaged group’s health information-seeking behaviour in Ghana. | 30 in-depth interviews (older adults with very low incomes, duration 40-45 minutes)  15 in-depth interviews (caregivers, duration 30-35 minutes)  15 in-depth interviews (formal healthcare providers, duration 30-35 minutes)  3 focus group discussions (only older adults with very low incomes, Each group discussion was comprised of 8–10 participants and lasted approximately 90 min) |
| Tingting Zhu 2019 | China | Quantitative descriptive studies / none | N = 434 (180 Male, 254 Female)  Age: ≥ 60 years (mean age [69.41 ± 6.45] years)  Health status: patients who met the diagnostic criteria of pre-diabetes ([ IFG 6.1 mmol/L ≤ fasting blood glucose <7.0  mmol/L] or [ IGT 7.8 mmol/L ≤ 2-hour postprandial blood glucose < 11.1 mmol/L]) | To investigate the status of diabetes information acquisition and its influencing factors among patients, 60 years and older, with pre-diabetes in rural China. | Diabetes information acquisition : the questionnaire of health literacy of diabetes of the public in China (Li L 2014) |
| Young Sam Oh 2018 | South Korea | Quantitative descriptive studies / comprehensive model of information  seeking (CMIS) | N = 235 (124 Male, 111 Female)  Age: ≥ 60 years (mean age 65.78 years)  Health status: not target specific disease populations | To examine the predictors of smartphone uses for health information–seeking (SHIS) in the Korean elderly. | Health literacy: health literacy scale (Centers for Disease Control and Prevention 2015) |
| Kristina Eriksson-Backa 2018 | Finland | Quantitative descriptive studies / none | N = 273 (121 Male, 152 Female)  Age: ≥ 65 years  Health status: not target specific disease populations | To examines older Finnish adults’ self-perceived capability to access, evaluate, understand and use health-related information, and their perceived ability to influence their health themselves, and the relationship between these factors and their current health, health behaviour and information seeking. | The questionnaire was developed by the researcher himself  Health information literacy: health information literacy scale (Eriksson-Backa 2012) |
| Xiaojing Sheng 2015 | USA | Quantitative descriptive studies / comprehensive model of information  seeking (CMIS) | N = 832 (349 Male, 483 Female)  Age: ≥ 60 years  Health status: not target specific disease populations | To examined some of these factors as identified in the comprehensive model of information seeking to find that demographics, trust in health information websites, perceived usefulness of the Internet, and internal locus of control each significantly impact seniors’ use of the Internet to seek health information. | The questionnaire was developed by the researcher himself  belief in internal locus of control: health locus of control scale (Wallston 1976)  Perceived health status: (Lumpkin 1989)  The information carrier characteristic variable(TRUST): (S. Wang 2004) |
| Kathryn E. Flynn 2006 | USA | Quantitative descriptive studies / none | N = 4528 (2174 Male, 2354 Female)  Age: ≥ 60 years  Health status: not target specific disease populations | To determine how patient characteristics are related to seeking health information online and to the timing of these searches in relation to doctor visits. | The questionnaire was developed by the researcher himself  health included the physical and mental component summary scores: 12-Item short-form health survey (Ware J 1996)  Personality: 5-factor model (McCrae R.R 2003) |
| Hendrika Meischke 2005 | USA | Quantitative descriptive studies / none | N = 318 (118 Male, 200 Female)  Age: ≥ 65 years (mean age 74 years)  Health status: not target specific disease populations | To investigate the demographic and psychosocial variables that are related to Internet access, health information seeking, and information seeking about heart attacks from the Internet among seniors. | The questionnaire was developed by the researcher himself |

Supplementary material 5 Barriers and facilitators derived from studies

| COM-B component | Themes | Frequency  (n= 46) | Barriers | Facilitators | Example quote(s)(Author and year) |
| --- | --- | --- | --- | --- | --- |
| Physical capability | 1.skills to access information (Facilitator) (Qualitative study data and Quantitative study data) | 3 |  | information retrieval capability | It is commonly believed that the elderly are limited in knowledge and skills, unable to search for information online, are not able to accurately assess or evaluate the obtained online health information, and lack self-belief in seeking information independently. However, one of the elderly respondents that we interviewed in our study had been independently seeking and buying diabetes drugs and consumables online for years. (Wenbo Xu 2024) |
|  |  |  | lack of skills in using the Internet |  | The most significant barrier to finding health information among older patients is "not being able to use the Internet". (Hasan Siamian 2024) |
|  |  |  | lack of information-seeking skills |  | "Good search skills help. I think it helps me find the information I need faster." (P6, P10, and P13). (Yufan Hu 2023)  Some older participants adopted a more passive attitude toward information-seeking for disease knowledge and did not need additional learning. (Yufan Hu 2023) |
|  | 2.age‑related physiological decline (Barrier) (Qualitative study data and Quantitative study data) | 2 | physiological degeneration |  | "After studying the rehabilitation videos posted by the rehabilitation doctor in the WeChat group, I invariably forget them quickly, and my recollection is worse than previously"(P7). (Yufan Hu 2023) |
|  |  |  |  | the comprehensibility of health information | The results of path analysis showed that the comprehensibility of health information had the largest direct effect on patients' health information seeking behavior, and indirectly affected patients' health information seeking behavior through the credibility of health information. (Peirong Cui 2022)  It may be related to the functional deterioration of cognition, hearing, and responsiveness in the elderly, which leads to a decline in the patient's ability to comprehend health information and severely hampers the patient's health information-seeking behaviour. (Peirong Cui 2022) |
|  | 3.healthy lifestyle habits (Facilitator) (Quantitative study data) | 2 |  | had regular exercise | Compared to the elderly who were nonhealth information seekers, the elderly having the SHIS experience (i.e., health information seekers) were younger, had higher education levels, had regular exercise, had higher levels of medical expenditure, and reported higher levels of health literacy. (Young Sam Oh 2018)  having SHIS was significantly correlated with lower age, higher education, regular exercise, higher medical expenditures, and higher health literacy. (Young Sam Oh 2018) |
|  |  |  | lifestyle types (the Uninterested Inactive) |  | Both lifestyle types influenced online health information significantly; that is, the Sociable Adventurer (β=.186, P<.001) and the Average Family Person (β=.160, P=.003) used online health information more often than the Uninterested Inactive. (Winja Weber 2020)  We found active and open-minded older adults (the Sociable Adventurer) as well as down-to-earth and family-oriented participants (the Average Family Person) to be more likely to seek online health information than uninterested, inactive ones (the Uninterested Inactive). This finding is in line with the results of another study indicating that people who are particularly curious and interested in many things use the internet more often for seeking health information. (Winja Weber 2020) |
| Psychological capability | 4.Limited ability to evaluate health information (Barrier) (Qualitative study data) | 1 |  | evaluation of obtained health information | The evaluation of the obtained health information by the elderly people included not only their evaluation of the online health information they obtained (for example, assessing the efficacy of a new drug), but also identifying complicated online information and eliminating bad information. (Wenbo Xu 2024) |
|  | 5.health literacy (Facilitator) (Quantitative study data) | 5 |  | health literacy | The results indicated that health literacy exerted a statistically significant direct influence on health information-seeking behavior at the 0.05 level, with a path coefficient of 0.89. The structural model accounted for forty-four percent of the variance in health literacy and sixty-three percent of the variance in health information-seeking behavior among older Muslim individuals. (Sumai Binbai 2025) |
|  |  |  |  | education level of high school or above | For the treatment stage, education level of high school or above (OR, 6.45; 95% CI, 1.34-30.98; p = 0.020) was positively associated with the high level of HISB. In the treatment stage, the education level of high school or above was positively associated with a high level of HISB, suggesting that the better-educated may have greater information literacy and awareness of the importance of seeking health information and actively engaging in discussions with healthcare providers. (Ya-Ping Wang 2025) |
|  |  |  |  | health literacy | The effect of health literacy on health information seeking behavior is obvious, and the effect size is second only to the intelligibility of health information, and it can also indirectly affect health information seeking behavior through the credibility of health information. (Peirong Cui 2022)  Health literacy is an important skill for patients to obtain health information. Previous studies have pointed out that health literacy is a controllable factor closely related to disease management skills. (Peirong Cui 2022) |
|  |  |  |  | health literacy | Individuals’ health literacy functions as a facilitator to enhance access to health and medical-related resources, such as healthcare services and medical information. Also, compared to people with lower health literacy, individuals with higher health literacy are more likely to understand various kinds of medical information (e.g., statistics related to medical issues), easily access information sources (e.g., websites related to medicine), and easily find the health information they seek. (Young Sam Oh 2018) |
|  |  |  |  | health information literacy | Health information literacy was significantly related to both interest in health information (chi-square=20.272, p≤.05) and seeking activity (chi-square=29.412, p≤.001). Less than half of those in the low health information literacy group, compared to 84% in the high health information literacy group were fairly or very interested. Regarding seeking activity, the difference was even more evident: 26% in the low group vs. 77% in the high group responded that they seek health information fairly or very often. (Kristina Eriksson-Backa 2018) |
|  | 6.trust in the source of information (Facilitator) (Qualitative study data, Quantitative study data and Mixed methods study data) | 6 | participants on anticoagulation |  | Participants ≥75 years old, as compared with those aged 65–74 years, and participants on anticoagulation, as compared to those who were not, were significantly less likely to seek health information online, after adjusting for a number of potentially confounding variables (adjusted odds ratio [aOR] = 0.55, 95% CI: 0.32–0.96; aOR = 0.62, 95% CI: 0.40–0.97, respectively). (Jordy Mehawej 2022)  In the present study, participants on anticoagulation therapy were less likely to engage in online health-seeking activities as compared with participants not on oral anticoagulants, which mainly involves a patient-physician shared decision-making process. (Jordy Mehawej 2022) |
|  |  |  | high self-reported efficacy in patient-physician interactions |  | Participants who sought health information online, compared with those who did not, were 32% less likely to report high efficacy in patient-physician interaction, after adjusting for several sociodemographic and clinical confounders (64% vs 72%, aOR = 0.68; 95% CI: 0.49–0.93). (Jordy Mehawej 2022)  This finding may reflect the possibility that patients who bring information found online to their clinic appointments have their physicians dismiss their information-seeking efforts and feel less confident to engage with their health care team going forward. Alternatively, it may be that patients with high perceived efficacy for patient-provider interactions advocate for themselves during medical visits, without the need to go online for their health-related information, and have fewer unanswered questions about their health than patients with lower perceived efficacy for patient-provider interactions. Future longitudinal research could examine whether low or reduced efficacy for patient-provider interactions prompts patients to look online to get information to answer their unanswered questions, or whether online health information seeking leads to lower perceived efficacy for communication, perhaps mediated by the provider’s response to the fruits of online searches. (Jordy Mehawej 2022) |
|  |  |  | trust in doctors |  | Furthermore, when it comes to health, the results suggest that the most frequently used and most trustworthy information source is the doctor. In particular, the older adults attribute medical competence only to the doctor; therefore, they put the decision about a treatment completely in the hands of the doctor. Trust is perceived as a particularly important determinant that arises from personal contact with the treating physician. (Sabine Theis 2021) |
|  |  |  | perceived poor attitude of healthcare providers/health workers |  | Most of the older adults with very low incomes interviewed stressed that healthcare providers should approach their work with professionalism and strengthen their interpersonal skills as they constantly talk to people who come for health information and treatment. From the FGD account, older adults with very low incomes argued that most of the nurses did not accord them with respect and had no time to listen to their health needs. (Williams Agyemang-Duah 2020)  "The way the nurses at public health facilities work is not professional. The nurses are worse when it comes to having time for patients. They are always in a hurry to finish and leave when we ask them questions about our health (A 73 -year female older adult with very low income, FGD)." (Williams Agyemang-Duah 2020) |
|  |  |  | communication/language problems |  | Almost all the older adults with very low incomes interviewed indicated that most of the doctors did not understand the local language (Twi) and could not speak it fluently which inhibited effective communication. They further explained that some of the health professionals speak English only. (Williams Agyemang-Duah 2020)  "Most of the doctors cannot speak Twi fluently so it is difficult for us to communicate with those who are not Akans. I cannot speak the English Language too. Because of the way they speak Twi, it is hard to comprehend what they say (A 70-year female older adult with very low income, in-depth interview)." (Williams Agyemang-Duah 2020) |
|  |  |  |  | trust in health information websites | The research found determinants of seniors’ use of the Internet for health information to be intention to use the Internet for health information, perceived usefulness of the Internet as an information source, trust in health information websites, age, and education level. (Xiaojing Sheng 2015)  Our CMIS “belief’ factor, internal locus of control, was found to negatively impact seniors’ use of the Internet for health information. (Xiaojing Sheng 2015) |
| Physical opportunity | 7.financial difficulties (Barrier) (Quantitative study data) | 3 |  | monthly income | For the pre-visit stage stage, a monthly income of CNY 2000-6000 (OR, 3.73; 95% CI, 1.08-12.91; p = 0.037) was positively associated with the high level of HISB. For the treatment stage, a monthly income of CNY 2000-6000 (OR, 0.13; 95% CI, 0.03-0.57; p = 0.007) was negatively associated with the high level of HISB. The observed shift in the association between a monthly income of CNY 2000–6000 and a high level of HISB from a positive correlation in the pre-visit stage to a negative correlation in the treatment stage revealed their focus and priorities change. It could imply that older adults within this specific income range might have different priorities or access to resources during treatment. (Ya-Ping Wang 2025) |
|  |  |  | financial reasons |  | The "barrier of lack of support in the family and financial matters" can limit the ability of older adults to access health information, which can also have negative consequences for their health outcomes. The observed shift in the association between a monthly income of CNY 2000–6000 and a high level of HISB from a positive correlation in the pre-visit stage to a negative correlation in the treatment stage revealed their focus and priorities change. It could imply that older adults within this specific income range might have different priorities or access to resources during treatment. (Hasan Siamian 2024) |
|  |  |  | patients of a lower socioeconomic status |  | Compared with women who did not use the internet to obtain health information, those who used the internet were younger (median age: 76 vs 81 years), more likely to be non-Hispanic white (90% [38,481/42,887] vs 87% [26,017/29,919]), earned a higher income (over $US 50,000: 55% [23,410/42,887] vs 33% [9991/29,919]), achieved a higher educational level (more than high school: 87% [37,493/42,887] vs 75% [22,377/29,919]), and were more likely to live with a partner (52% [22,457/42,887] vs 36% [10,759/29,919]) (all P<.001). (Mina S Sedrak 2020)  Disparities in online health information–seeking exist among patients who are ethnic minorities and of a lower socioeconomic status. Consistent with prior studies, our findings add to the evidence that digital health is not reaching all seniors equally. (Mina S Sedrak 2020) |
|  | 8.credibility of accessible health information (Facilitator) (Quantitative study data) | 1 | lower credibility of accessible health information |  | The level of health information-seeking behaviour was worse among patients with low educational attainment, lower credibility of accessible health information, living in rural areas, low monthly income, and the type of health insurance being the new rural cooperative medical insurance. This is related to the fact that those with low educational attainment have greater barriers to accessing health information, the dramatic increase in the amount of information leads to a lower level of credibility of health information, health information resources accessible to rural residents are more scarce, and those with low monthly income and new rural cooperative medical insurance have a higher healthcare burden. (Peirong Cui 2022) |
|  | 9.access to information sources (Facilitator) (Qualitative study data, Quantitative study data and Mixed methods study data) | 5 |  | living in rural areas | For the follow-up stage, living in rural areas (OR, 8.18; 95% CI, 1.15-57.91; p = 0.035) was facilitating factors associated with the HISB. Rural areas often lack convenient access to professional eye care services, and the follow-up stage requires continuous monitoring and management of the condition. This means that rural-dwelling older adults might rely more on self-initiated information-seeking. (Ya-Ping Wang 2025) |
|  |  |  | not having Internet access |  | For the pre-visit stage stage, not having Internet access (OR, 0.17; 95% CI, 0.04-0.68; p = 0.013) was negatively associated with the high level of HISB. (Ya-Ping Wang 2025) |
|  |  |  |  | health information scanning | The results showed a signiﬁcant positive association (β =.65, p <.001). Respondents who scanned more health information on WeChat also reported a higher frequency of seeking health information on this platform. (Wenshu Li 2024) |
|  |  |  | disease and health information service system needs to be improved |  | While some participants could search for health information using the disease health services system, we identified a gap between the needs of stroke survivors and the system’s design. (Yufan Hu 2023)  Other participants noted that features of their health information search site, such as the site’s font being too small and the color of links not being prominent, were barriers to efficiently searching for health information. (Yufan Hu 2023) |
|  |  |  |  | lack of information sources | The results indicate that health issues and symptoms initiate the information search. Furthermore, if the principal information source—the doctor—does not provide enough information, search activities are initiated. Older adults’ information behavior can thus be considered occasional. Occasional searching could be an alternative explanation for the heterogeneous need for general information. (Sabine Theis 2021)  The need for information coming directly from the doctor and more intensive communication between doctors and patients reveals the fundamental importance of doctors for older patients. Despite the doctors’ position as the most trustworthy source of information, older people see their lack of time as a barrier to having their information needs met. (Sabine Theis 2021) |
| Social opportunity | 10.family support (Facilitator) (Qualitative study data) | 3 |  | spousal caregiver and child caregiver | For the follow-up stage, spousal caregiver (OR, 6.36; 95% CI, 1.14-35.40; p = 0.035) and child caregiver (OR, 10.64; 95% CI, 2.14-53.02; p = 0.004) were facilitating factors associated with the HISB. Spousal or child caregivers play an active role in facilitating information acquisition. This finding emphasizes the significance of considering both environmental and social support factors in understanding and promoting HISB among older adults with VI, especially those in the crucial follow-up phase of their eye care journey. (Ya-Ping Wang 2025) |
|  |  |  |  | emotional needs (family affection, with a close or estranged relationship between the elderly and their family) | We found that the elderly would discuss their health status with their family members in order to receive more attention from them (offspring), and they were able to obtain care and support from their family through such interactions. (Wenbo Xu 2024)  Other elderly people improved care-related interactions with their family members by sharing their opinions on the use of health products purchased by their family as a proxy. (Wenbo Xu 2024) |
|  |  |  | children played a weaker role |  | Regarding the type of people for social support, children were the most desired caregiver group reported by participants. Nevertheless, patients were less likely to mention how their children helped them access health information independently. (Yufan Hu 2023)  "The kids work abroad and can only come home during holidays, and they hired caregivers to take care of me." (P4, P9, and P10). (Yufan Hu 2023) |
|  | 11.social support (Facilitator) (Quantitative study data) | 2 |  | health opinion leadership | The results revealed that health opinion leadership was positively associated with both health information seeking (β =.10, p <.05). Respondents who identiﬁed themselves as health opinion leaders were more inclined to pay attention to health information they encountered on WeChat and actively seek health information on this platform. (Wenshu Li 2024) |
|  |  |  |  | social support | Social support influenced online health information seeking directly (β = .23, p  < .05) and indirectly through self-efficacy (β = .03, p  < .05). (Yaru Zhu 2023)  The care and support of family members prompted them to use internet resources effectively and actively participate in disease management and health maintenance. (Yaru Zhu 2023) |
| Reflective motivation | 12.self-efficacy (Facilitator) (Qualitative study data and Quantitative study data) | 5 |  | self-efficacy | We found most elderly people with chronic illnesses had limited knowledge, skills, and low levels of literacy pertaining to electronic health information, and lacked self-belief in seeking online health information independently. (Wenbo Xu 2024) |
|  |  |  |  | efficacy | The results revealed that respondents reported a higher level of efﬁcacy were likely to engage in more frequent health information seeking (β =.09,p <.05). (Wenshu Li 2024) |
|  |  |  |  | self-efficacy | Self-efficacy directly influenced online health information seeking (β = .26, p  < .05). (Yaru Zhu 2023)  People believing in their ability to search and select high-quality health information are likely to find the internet useful, thus, facilitating their participation in online health information seeking. Emotional support from family members is critical to increase their confidence in disease treatment. (Yaru Zhu 2023) |
|  |  |  |  | self-efficacy | Self-efficacy, perceived encouragement, positive attitude toward the e-HISB, perceived usefulness, and challenges of being visited by physicians were statistically significant and increased the odds of e-HISB by 37.00%, 22.00%, 56.00%, 31.00%, and 6.00%, respectively. Conversely, perceived barriers to e-HISB reduced the odds by 17.00%. Additionally, after adjusting for the demographic variables, self-efficacy, positive attitude toward the e-HISB, perceived usefulness, and perceived barriers to e-HISB remained statistically significant; specifically, the first 3 increased the odds by 12.00%, 24.00%, and 15.00%. (Sara Pourrazavi 2022)  Self-efficacy, which is considered an essential component of accomplishment and well-being, has consistently been identified as an important determinant of health behavior in older adults. In fact, self-efficacy has theoretical and practical implications in enhancing the health status of older adults; that is, people with strong beliefs in their ability to achieve health-related goals are capable of strengthening their efforts to overcome barriers and improve their health condition. (Sara Pourrazavi 2022) |
|  |  |  |  | self-efficacy | Self-efficacy significantly positively affects patients' health information seeking behavior, which accords with Bandura's self-efficacy theory. The reason may be that the more confident patients are about disease management, the more inclined patients are to access health information to promote health. (Peirong Cui 2022) |
|  | 13.perceived usefulness of health information (Facilitator) (Quantitative study data and Qualitative study data) | 4 |  | perceived usefulness | Self-efficacy, perceived encouragement, positive attitude toward the e-HISB, perceived usefulness, and challenges of being visited by physicians were statistically significant and increased the odds of e-HISB by 37.00%, 22.00%, 56.00%, 31.00%, and 6.00%, respectively. Conversely, perceived barriers to e-HISB reduced the odds by 17.00%. Additionally, after adjusting for the demographic variables, self-efficacy, positive attitude toward the e-HISB, perceived usefulness, and perceived barriers to e-HISB remained statistically significant; specifically, the first 3 increased the odds by 12.00%, 24.00%, and 15.00%, respectively, while perceived barriers reduced the odds by 16.00%. (Sara Pourrazavi 2022)  It is shown that older adults consider the Internet as a valuable tool for accessing the information they need. Moreover, it is documented that obtaining health information through the Internet can increase older adults’ positive emotions and satisfaction , especially among those who are homebound, live in remote areas, and cannot easily travel. (Sara Pourrazavi 2022) |
|  |  |  |  | positive attitude toward the e-HISB | Self-efficacy, perceived encouragement, positive attitude toward the e-HISB, perceived usefulness, and challenges of being visited by physicians were statistically significant and increased the odds of e-HISB by 37.00%, 22.00%, 56.00%, 31.00%, and 6.00%, respectively. Conversely, perceived barriers to e-HISB reduced the odds by 17.00%. Additionally, after adjusting for the demographic variables, self-efficacy, positive attitude toward the e-HISB, perceived usefulness, and perceived barriers to e-HISB remained statistically significant; specifically, the first 3 increased the odds by 12.00%, 24.00%, and 15.00%, respectively, while perceived barriers reduced the odds by 16.00%. (Sara Pourrazavi 2022)  The formation of a positive attitude toward the e-HISB among older adults with the increased popularity of the Internet and understanding its usefulness may be instrumental for e-HISB. (Sara Pourrazavi 2022) |
|  |  |  | inadequate knowledge about the benefits of seeking health information |  | It was observed that older adults with very low incomes did have little knowledge about the importance of accessing health information from healthcare providers, particularly doctors and nurses, at their age. (Williams Agyemang-Duah 2020)  "Why should I seek health information from doctors when I can get health information from my friends or relatives and buy medicines at drug stores? I do not need to see a doctor for normal symptoms like headaches, and feeling feverish. For we the people in rural areas, we are not fond of seeking health information from healthcare providers (A 73-year female older adult with very low income, in-depth interview)." (Williams Agyemang-Duah 2020) |
|  |  |  |  | perceived usefulness of the Internet as an information source | The research found determinants of seniors’ use of the Internet for health information to be intention to use the Internet for health information, perceived usefulness of the Internet as an information source, trust in health information websites, age, and education level. (Xiaojing Sheng 2015)  This research also found that trust in health information websites and perceived usefulness of the Internet positively affected intention and that trust and perceived importance of health information access on the Internet positively affected perceived usefulness of the Internet. (Xiaojing Sheng 2015) |
|  | 14.intention to maintain health status (Facilitator) (Qualitative study data, Quantitative study data and Mixed methods study data) | 11 |  | perception of health status | The findings indicated that health perception exerted a statistically significant direct influence on health literacy at the 0.05 level, with a path coefficient of 0.63. Nonetheless, health perception did not have a substantial direct impact on health information-seeking behavior. Health perception indirectly influences health information-seeking behavior. The research indicated that health perception had a statistically significant indirect influence on health information-seeking behavior via health literacy, with a path coefficient of 0.56. (Sumai Binbai 2025) |
|  |  |  |  | health status | For the pre-visit stage stage, poor self-reported overall health (OR, 5.10; 95% CI, 1.46-17.90; p = 0.011) were positively associated with the high level of HISB. For the treatment stage, poor self-reported overall health (OR, 7.68; 95% CI, 1.45-40.64; p = 0.017) was positively associated with the high level of HISB. For the follow-up stage, general (OR, 13.74; 95% CI, 1.89-99.79; p = 0.010) and poor (OR, 67.81; 95% CI, 9.73-472.69; p < 0.001) self-reported overall health were facilitating factors associated with the HISB. Those who perceive their health as poor are more likely to be actively engaged in information-seeking activities in an attempt to better understand, manage, and potentially improve their situation. (Ya-Ping Wang 2025)  For the treatment stage, bilateral visual impairment (OR, 0.16; 95% CI, 0.03-0.81; p = 0.026), mild visual impairment (OR, 0.11; 95% CI, 0.02-0.62; p = 0.013), and moderate to blindness (OR, 0.16; 95% CI, 0.03-0.81; p = 0.026) in the better eye were negatively associated with the high level of HISB. (Ya-Ping Wang 2025) |
|  |  |  |  | perception of their own health and concern about one’s health | The elderly people with one or more chronic illnesses appeared well aware of their health status, attached great importance to online health information, and desired family affection in their daily life. Conversely, most elderly people in good health status did not give much thought to online health information, let alone understand its significance. (Wenbo Xu 2024)  The elderly with proxy seeking had better knowledge about their health needs (such as rehabilitation devices, drugs, consumables, and so on). Concurrently, the elderly who knew their health needs well were more alert to changes in their health status brought about by rehabilitation devices and drugs. (Wenbo Xu 2024) |
|  |  |  |  | patient activation | Patient activation influenced online health information seeking directly (β = .39, p  < .05) and indirectly through self-efficacy (β = .17, p  < .05). (Yaru Zhu 2023)  Patients with high activation level attach importance to their role and status in disease management, are willing to communicate with health care staff, actively seek health information and purposefully search for disease-related information. On the other hand, patients with lower levels of activation likely feel powerless to treat their disease, thus, are less likely to actively seek information about the disease. (Yaru Zhu 2023)  When patients are actively involved in disease management and keep abreast of their health status, their confidence in the cure of the disease increases. In addition, patient activation plays an essential role in acquiring knowledge of CHD. These results further suggest that increased patient activation can promote online health information seeking, and improve their quality of life. (Yaru Zhu 2023) |
|  |  |  |  | lower atrial fibrillation-related quality of life | Participants who sought health information online, compared with those who did not, were significantly more likely to have a lower AF-related quality of life, after adjusting for several potentially confounding factors (44% vs 37%, aOR = 1.56; 95% CI: 1.15–2.13). (Jordy Mehawej 2022)  Clinicians should provide patients with atrial fibrillation who seek health information online a list of online resources and enhanced educational handouts that have been shown to improve patient understanding and reduce unnecessary health-seeking behaviors, thereby improving the quality of life of patients with atrial fibrillation. (Jordy Mehawej 2022) |
|  |  |  |  | health issues and symptoms | The results indicate that health issues and symptoms initiate the information search. Furthermore, if the principal information source-the doctor-does not provide enough information, search activities are initiated. Older adults’ information behavior can thus be considered occasional. Occasional searching could be an alternative explanation for the heterogeneous need for general information. (Sabine Theis 2021) |
|  |  |  |  | health status (healthy seniors) | Educational status (β=.126, P=.002) and health status (β=.081, P=.04) correlated positively and significantly with online health information use, indicating that highly educated and healthy seniors tend to use online health information more often than less well-educated subjects and those with poorer self-reported health. (Winja Weber 2020) |
|  |  |  |  | history of hyperglycaemia | We found that the elderly subjects with pre-diabetes who were older (B=−1.948, p=0.034), had a lower educational level (B=2.541, p=0.005) and did not have a history of hyperglycaemia (B=7.671, p=0.000) were more likely to have poorer ability to access information on diabetes. (Tingting Zhu 2019) |
|  |  |  |  | intention to use the Internet for health information | The research found determinants of seniors’ use of the Internet for health information to be intention to use the Internet for health information, perceived usefulness of the Internet as an information source, trust in health information websites, age, and education level. (Xiaojing Sheng 2015)  This research also found that trust in health information websites and perceived usefulness of the Internet positively affected intention and that trust and perceived importance of health information access on the Internet positively affected perceived usefulness of the Internet. (Xiaojing Sheng 2015) |
|  |  |  |  | history of acute myocardial infarction | Participants with a history of acute myocardial infarction (33% vs 8%, P < .05), with a family history of acute myocardial infarction (75% vs 43%, P < .06), and who were older (77 vs 71 years of age, P < .03) were more likely to report seeking information on heart attacks in the univariate analyses. (Hendrika Meischke 2005)  A history of acute myocardial infarction was almost significant. This suggests that only the strongest cues become motivators for seeking specific health information on the Internet. (Hendrika Meischke 2005) |
|  |  |  |  | family history of acute myocardial infarction | Family history of acute myocardial infarction and education were significantly related (P < .05) to use of Internet for health information. Those with a family history and with higher levels of education more frequently reported seeking health information from the Internet than those who did not. (Hendrika Meischke 2005)  Seniors with a family history were more likely to report searching the web for health information than those without such a history, suggesting that the presence of health problems in one’s direct environment may trigger health information seeking from the Internet. (Hendrika Meischke 2005) |
| Automatic motivation | 15.personality trait (Mixed) (Quantitative study data) | 1 |  | openness-to-experience | Years of education (odds ratio [OR]=1.09, confidence interval [CI]=1.06 to 1.13) and openness-to-experience (OR=1.26, CI=1.16 to 1.36) were positively associated with searching online for health information irrespective of timing in relation to doctor visits. (Kathryn E. Flynn 2006) |

Supplementary material 6 The Behaviour Change Wheel

Supplementary material 7 Definitions of COM-B model components and examples

| COM-B model component | Definition | Example |
| --- | --- | --- |
| Physical capability | Physical skill, strength or stamina | Having the skill to take a blood sample |
| Psychological capability | Knowledge or psychological skills, strength or stamina to engage in the necessary mental processes | Understanding the impact of CO_2_ on the environment |
| Physical opportunity | Opportunity afforded by the environment involving time, resources, locations, cues, physical 'affordance' | Being able to go running because one owns appropriate shoes |
| Social opportunity | Opportunity afforded by interpersonal influences, social cues and cultural normsthat influence the way that we think about things, e.g. the words and concepts that make up our language | Being able to smoke in the house of someone who smokes but not in the middle of a boardroom meeting |
| Reflective motivation | Reflective processes involving plans (self-conscious intentions) and evaluations (beliefs about what is good and bad) | Intending to stop smoking |
| Automatic motivation | Automatic processes involving emotional reactions, desires (wants and needs), impulses, inhibitions, drive states and reflex responses | Feeling anticipated pleasure at the prospect of eating a piece of chocolate cake |

**Supplementary material 8 Mixed Methods Appraisal Tool (MMAT) 2018**

| **Category of study designs** | **Methodological quality criteria** |
| --- | --- |
| Screening questions (for all types) | S1. Are there clear research questions? |
|  | S2. Do the collected data allow to address the research questions? |
| 1. Qualitative | 1.1. Is the qualitative approach appropriate to answer the research question? |
|  | 1.2. Are the qualitative data collection methods adequate to address the research questions? |
|  | 1.3. Are the findings adequately derived from the data? |
|  | 1.4. Is the interpretation of results sufficiently substantiated by data? |
|  | 1.5. Is there coherence between qualitative data sources, analysis and interpretation? |
| 2. Quantitative randomized controlled trials | 2.1. Is randomization appropriately performed? |
|  | 2.2. Are the groups comparable at baseline? |
|  | 2.3. Are there complete outcome data? |
|  | 2.4. Are outcome assessors blinded to the intervention provided? |
|  | 2.5 Did the participants adhere to the assigned intervention? |
| 3. Quantitative non-randomize | 3.1. Are the participants representative of the target population? |
|  | 3.2. Are measurements appropriate regarding both the outcome and intervention (or exposure)? |
|  | 3.3. Are there complete outcome data? |
|  | 3.4. Are the confounders accounted for in the design and analysis? |
|  | 3.5. During the study period, is the intervention administered (or exposure occurred) as intended? |
| 4. Quantitative descriptive | 4.1. Is the sampling strategy relevant to address the research question? |
|  | 4.2. Is the sample representative of the target population? |
|  | 4.3. Are the measurements appropriate? |
|  | 4.4. Is the risk of nonresponse bias low? |
|  | 4.5. Is the statistical analysis appropriate to answer the research question? |
| 5. Mixed methods | 5.1. Is there an adequate rationale for using a mixed methods design to address the research question? |
|  | 5.2. Are the different components of the study effectively integrated to answer the research question? |
|  | 5.3. Are the outputs of the integration of qualitative and quantitative components adequately interpreted? |
|  | 5.4. Are divergences and inconsistencies between quantitative and qualitative results adequately addressed? |
|  | 5.5. Do the different components of the study adhere to the quality criteria of each tradition of the methods involved? |

**Supplementary material 9 PRISMA 2020 Checklist**

| **Section and Topic** | **Item #** | **Checklist item** | **Location where item is reported** |
| --- | --- | --- | --- |
| **TITLE** | | |  |
| Title | 1 | Identify the report as a systematic review. | Page 1 |
| **ABSTRACT** | | |  |
| Abstract | 2 | See the PRISMA 2020 for Abstracts checklist. | Page 1, 2 |
| **INTRODUCTION** | | |  |
| Rationale | 3 | Describe the rationale for the review in the context of existing knowledge. | Page 2, 3 |
| Objectives | 4 | Provide an explicit statement of the objective(s) or question(s) the review addresses. | Page 3 |
| **METHODS** | | |  |
| Eligibility criteria | 5 | Specify the inclusion and exclusion criteria for the review and how studies were grouped for the syntheses. | Page 4, 5 |
| Information sources | 6 | Specify all databases, registers, websites, organisations, reference lists and other sources searched or consulted to identify studies. Specify the date when each source was last searched or consulted. | Page 5 and Supplementary material 2 |
| Search strategy | 7 | Present the full search strategies for all databases, registers and websites, including any filters and limits used. | Page 5 and Supplementary material 1 |
| Selection process | 8 | Specify the methods used to decide whether a study met the inclusion criteria of the review, including how many reviewers screened each record and each report retrieved, whether they worked independently, and if applicable, details of automation tools used in the process. | Page 5 |
| Data collection process | 9 | Specify the methods used to collect data from reports, including how many reviewers collected data from each report, whether they worked independently, any processes for obtaining or confirming data from study investigators, and if applicable, details of automation tools used in the process. | Page 5 |
| Data items | 10a | List and define all outcomes for which data were sought. Specify whether all results that were compatible with each outcome domain in each study were sought (e.g. for all measures, time points, analyses), and if not, the methods used to decide which results to collect. | Page 5 |
|  | 10b | List and define all other variables for which data were sought (e.g. participant and intervention characteristics, funding sources). Describe any assumptions made about any missing or unclear information. | Page 5 |
| Study risk of bias assessment | 11 | Specify the methods used to assess risk of bias in the included studies, including details of the tool(s) used, how many reviewers assessed each study and whether they worked independently, and if applicable, details of automation tools used in the process. | Page 5 |
| Effect measures | 12 | Specify for each outcome the effect measure(s) (e.g. risk ratio, mean difference) used in the synthesis or presentation of results. | NA |
| Synthesis methods | 13a | Describe the processes used to decide which studies were eligible for each synthesis (e.g. tabulating the study intervention characteristics and comparing against the planned groups for each synthesis (item #5)). | NA |
|  | 13b | Describe any methods required to prepare the data for presentation or synthesis, such as handling of missing summary statistics, or data conversions. | Page 6 |
|  | 13c | Describe any methods used to tabulate or visually display results of individual studies and syntheses. | Page 6 |
|  | 13d | Describe any methods used to synthesize results and provide a rationale for the choice(s). If meta-analysis was performed, describe the model(s), method(s) to identify the presence and extent of statistical heterogeneity, and software package(s) used. | Page 6 |
|  | 13e | Describe any methods used to explore possible causes of heterogeneity among study results (e.g. subgroup analysis, meta-regression). | NA |
|  | 13f | Describe any sensitivity analyses conducted to assess robustness of the synthesized results. | NA |
| Reporting bias assessment | 14 | Describe any methods used to assess risk of bias due to missing results in a synthesis (arising from reporting biases). | NA |
| Certainty assessment | 15 | Describe any methods used to assess certainty (or confidence) in the body of evidence for an outcome. | Page 6 |
| **RESULTS** | | |  |
| Study selection | 16a | Describe the results of the search and selection process, from the number of records identified in the search to the number of studies included in the review, ideally using a flow diagram. | Page 7 |
|  | 16b | Cite studies that might appear to meet the inclusion criteria, but which were excluded, and explain why they were excluded. | NA |
| Study characteristics | 17 | Cite each included study and present its characteristics. | Page 7 and Supplementary material 4 |
| Risk of bias in studies | 18 | Present assessments of risk of bias for each included study. | Page 8 and Supplementary material 3 |
| Results of individual studies | 19 | For all outcomes, present, for each study: (a) summary statistics for each group (where appropriate) and (b) an effect estimate and its precision (e.g. confidence/credible interval), ideally using structured tables or plots. | Page 9 and Supplementary material 5 |
| Results of syntheses | 20a | For each synthesis, briefly summarise the characteristics and risk of bias among contributing studies. | Page 9 |
|  | 20b | Present results of all statistical syntheses conducted. If meta-analysis was done, present for each the summary estimate and its precision (e.g. confidence/credible interval) and measures of statistical heterogeneity. If comparing groups, describe the direction of the effect. | Page 9 |
|  | 20c | Present results of all investigations of possible causes of heterogeneity among study results. | NA |
|  | 20d | Present results of all sensitivity analyses conducted to assess the robustness of the synthesized results. | NA |
| Reporting biases | 21 | Present assessments of risk of bias due to missing results (arising from reporting biases) for each synthesis assessed. | NA |
| Certainty of evidence | 22 | Present assessments of certainty (or confidence) in the body of evidence for each outcome assessed. | NA |
| **DISCUSSION** | | |  |
| Discussion | 23a | Provide a general interpretation of the results in the context of other evidence. | Page 10, 11 |
|  | 23b | Discuss any limitations of the evidence included in the review. | Page 14 |
|  | 23c | Discuss any limitations of the review processes used. | Page 14 |
|  | 23d | Discuss implications of the results for practice, policy, and future research. | Page 13 |
| **OTHER INFORMATION** | | |  |
| Registration and protocol | 24a | Provide registration information for the review, including register name and registration number, or state that the review was not registered. | Page 4 |
|  | 24b | Indicate where the review protocol can be accessed, or state that a protocol was not prepared. | Page 4 |
|  | 24c | Describe and explain any amendments to information provided at registration or in the protocol. | NA |
| Support | 25 | Describe sources of financial or non-financial support for the review, and the role of the funders or sponsors in the review. | Page 15 |
| Competing interests | 26 | Declare any competing interests of review authors. | Page 15 |
| Availability of data, code and other materials | 27 | Report which of the following are publicly available and where they can be found: template data collection forms; data extracted from included studies; data used for all analyses; analytic code; any other materials used in the review. | Supplementary materials |

*From:* Page MJ, McKenzie JE, Bossuyt PM, Boutron I, Hoffmann TC, Mulrow CD, et al. The PRISMA 2020 statement: an updated guideline for reporting systematic reviews. BMJ 2021;372:n71. doi: 10.1136/bmj.n71. This work is licensed under CC BY 4.0. To view a copy of this license, visit <https://creativecommons.org/licenses/by/4.0/>
